# A Bilayer Microarray Patch (MAP) for HIV Pre-Exposure Prophylaxis: The Role of MAP Designs and Formulation Composition in Enhancing Long-Acting Drug Delivery

**DOI:** 10.3390/pharmaceutics16010142

**Published:** 2024-01-20

**Authors:** Lalitkumar K. Vora, Ismaiel A. Tekko, Fabiana Volpe Zanutto, Akmal Sabri, Robert K. M. Choy, Jessica Mistilis, Priscilla Kwarteng, Courtney Jarrahian, Helen O. McCarthy, Ryan F. Donnelly

**Affiliations:** 1 Medical Biology Centre, School of Pharmacy, Queen’s University Belfast, 97 Lisburn Road, Belfast BT9 7BL, UK; l.vora@qub.ac.uk (L.K.V.); i.tekko@qub.ac.uk (I.A.T.); f.volpezanutto@qub.ac.uk (F.V.Z.); a.binsabri@qub.ac.uk (A.S.);; 2 PATH, 2201 Westlake Avenue, Seattle, DC 98121, USA; rchoy@path.org (R.K.M.C.); jmistilis@path.org (J.M.);

**Keywords:** cabotegravir, dissolving microarray patch, microneedles, HIV, long-acting drugs, pre-exposure prophylaxis, intradermal delivery

## Abstract

Microarray patches (MAPs) have shown great potential for efficient and patient-friendly drug delivery through the skin; however, improving their delivery efficiency for long-acting drug release remains a significant challenge. This research provides an overview of novel strategies aimed at enhancing the efficiency of MAP delivery of micronized cabotegravir sodium (CAB Na) for HIV pre-exposure prophylaxis (PrEP). The refinement of microneedle design parameters, including needle length, shape, density, and arrangement, and the formulation properties, such as solubility, viscosity, polymer molecular weight, and stability, are crucial for improving penetration and release profiles. Additionally, a bilayer MAP optimization step was conducted by diluting the CAB Na polymeric mixture to localize the drug into the tips of the needles to enable rapid drug deposition into the skin following MAP application. Six MAP designs were analyzed and investigated with regard to delivery efficiency into the skin in ex vivo and in vivo studies. The improved MAP design and formulations were found to be robust and had more than 30% in vivo delivery efficiency, with plasma levels several-fold above the therapeutic concentration over a month. Repeated weekly dosing demonstrated the robustness of MAPs in delivering a consistent and sustained dose of CAB. In summary, CAB Na MAPs were able to deliver therapeutically relevant levels of drug.

## 1. Introduction

HIV continues to be a major cause of morbidity and mortality worldwide; more than 76 million people have been infected, culminating in more than 33 million deaths globally since the start of this epidemic in the 1980s [1,2,3,4]. A significant tool to reduce HIV infections is the use of pre-exposure prophylaxis (PrEP) to deliver an antiretroviral (ARV) to individuals at substantial risk of HIV infection. In 2021, an estimated 1.6 million people received oral PrEP (daily dose) at least once [5]. 

Despite the effectiveness of oral PrEP, daily administration may impose a considerable burden on patients, resulting in poor compliance and therefore a persistent risk of HIV acquisition [6]. Because of this limitation, there is impetus to develop a long-acting ARV formulation to simplify the overall prevention regimen and thus increase overall patient compliance. Dedicated research within the field of pharmaceutical science led to the approval of Apretude^®^ (ViiV Healthcare), a long-acting injectable formulation of the integrase strand transfer inhibitor cabotegravir (CAB). The intramuscular (IM) injection is given in-clinic every 2 months to reduce the risk of HIV infection [7]. Additionally, a monthly dapivirine vaginal ring has been approved for the prevention of HIV in several countries [8], and other regimens, such as a twice-yearly subcutaneous injection of lenacapavir, have been recently approved [9]. Long-acting drug delivery technologies such as implants are also under development for HIV PrEP [10,11,12,13].

Injectable formulations must be delivered by skilled health care professionals, adding to the cost of PrEP and increasing the burden on health care programs, particularly in areas with fewer health care workers. This is a major global health issue, as the HIV incidence rate is exceptionally high in sub-Saharan Africa relative to other parts of the world [14]. These routine, painful injections may also have a negative effect on patient compliance over the long term. Therefore, there is a need to refine the way PrEP is administered to increase accessibility. One approach is to reformulate ARVs for delivery via microarray patches (MAPs), also known as microneedle patches [15,16,17]. These are patches with micron-size projections on a flat baseplate, which upon application to the skin, in a manner similar to the use of a bandage, generate microchannels for drug delivery. In general, MAPs can be classified into three main categories based on their mode of delivering payload into the skin: dissolving, hydrogel-forming, and coated MAPs. Dissolving MAPs have been the most widely explored and studied with respect to the delivery of ARVs [18].

Compared to the conventional IM injection of ARV formulations, MAPs can be applied in a minimally invasive fashion to the skin to achieve systemic delivery of ARVs. Despite the ease and simplicity of applying these patches, the insertion and delivery of the payload via MAP involve complex factors such as design and needle tip composition, as well as the overall physiochemical properties of the delivered active pharmaceutical ingredient [19,20]. Therefore, it would be judicious to meticulously consider these crucial factors when designing a MAP formulation for the delivery of ARVs. In addition, these MAPs ought to be able to achieve high drug loading and be capable of delivering the active pharmaceutical ingredient with acceptable delivery efficiency into the skin to serve as an intradermal depot for sustained delivery of hydrophobic particulate payload overall for several weeks. Guided by these formulation goals and the purpose of developing a minimally invasive MAP strategy to deliver ARVs, this paper summarizes the different strategies that have been explored with the aim of augmenting the efficiency of MAPs for the long-acting delivery of CAB sodium (CAB Na) for HIV PrEP. 

## 2. Materials and Methods

### 2.1. Materials

Long-acting cabotegravir (CAB LA) nanosuspension vials for IM injection with drug content (400 mg/2 mL) and micronized powders of CAB Na were supplied by ViiV Healthcare (Research Triangle Park, NC, USA). Poly(vinyl alcohol), or PVA, of molecular weight (MW) 9 to 10 kDa, 80% hydrolyzed (PVA 10K), and of MW 31 to 50 kDa, 87% to 89% hydrolyzed (PVA 50K), were purchased from Sigma–Aldrich (Dorset, UK). Poly(vinyl pyrrolidone) K29-32 gel (MW 58 kDa) was a gift from Ashland (Kidderminster, UK). Liquid silicone elastomer mix was purchased from Nusil Technology (Buckinghamshire, UK). Ultrapure water was obtained from a water purification system (Elga PURELAB^®^ DV 25, Veolia Water Systems, Dublin, Ireland). All other chemicals and materials were of analytical reagent grade and supplied by Sigma–Aldrich.

### 2.2. Strategies to Improve MAP Delivery Efficiency via Refinement of MAP Designs

The MAP design parameters, including needle length, shape, density, and arrangement, are crucial for improving various aspects of microneedle performance, such as penetration efficiency, drug distribution within the skin, and drug release profiles. Six MAPs of various geometries and shapes (Table 1) were designed and manufactured for use in the investigation of various strategies to improve CAB intradermal delivery efficiency [21,22].

**Figure 1:**
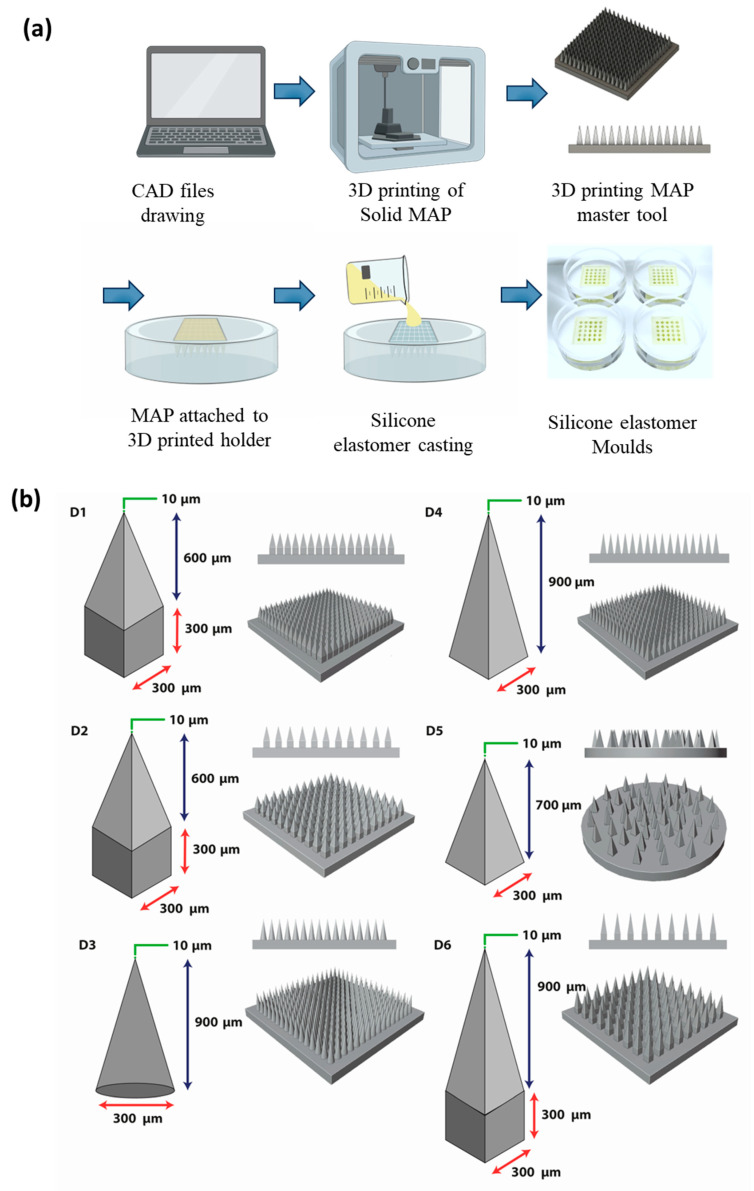
(**a**) Schematic illustrating the fabrication and manufacture of MAP molds via two-photon polymerization 3D printing. (**b**) MAP array designs developed for two-photon polymerization 3D printing (except D5).

Computer-aided design was employed to create the various MAP designs. These designs were then used to generate a primary template (Figure 1a) through a process called two-photon polymerization of IP-S resins, which are acrylate-based polymers. This process was carried out using a Photonic Professional GT 3D printer system from Nanoscribe GmbH (Leopoldshafen, Germany), as detailed in our previous research [23]. Next, each MAP design’s primary template was affixed to a 3D-printed (UltiMaker B.V., Geldermalsen, Germany) holder made of polylactic acid and utilized to fabricate female molds for the MAPs. These molds were produced using a transparent silicone/elastomer mixture (Figure 1a). To eliminate any remaining air bubbles, the silicone-filled templates underwent centrifugation at 3500 rpm for 5 min, followed by curing at 60 °C for a period of 3 to 6 h. Once the primary template cooled down, the PDMS mold (Figure 1b) was extracted. This PDMS mold served as the main component for manufacturing the bilayer CAB Na MAPs and could be reused multiple times. 

### 2.3. Strategies to Enhance MAP Delivery Efficiency via Fabrication of Improved Bilayer MAPs

**Figure 2:**
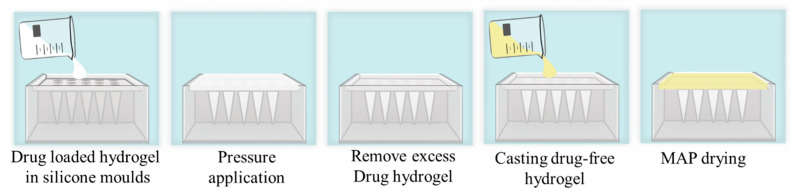
Schematic representation of the bilayer MAP preparation method.

Bilayer CAB Na MAPs were prepared from various CAB Na-loaded hydrogels (to form the first MAP layer) and drug-free hydrogels (to form the second MAP layer) using a positive pressure chamber by either two-step or three-step casting as needed, as depicted in Figure 2 and previously described [24].

Briefly, 100 mg of the finalized CAB Na-loaded hydrogel was dispensed onto the PDMS mold of the developed MAP and then placed in the positive pressure chamber at 5 bars for 3 min. The PDMS mold was removed from the chamber, excess drug-loaded hydrogel was carefully scraped using a stainless steel spatula, and the mold was immediately returned to the chamber at 5 bar pressure for 15 min. Subsequently, the PDMS mold was removed from the chamber, and the first MAP layer was dried overnight at room temperature. The second MAP layer was then prepared by pouring the optimized drug-free polymeric blend on top of the first layer and placing the mold in a positive pressure chamber at 5 bar for 15 min. Finally, the bilayer CAB Na MAP was dried at room temperature for 24 h and at 37 °C for 24 h before being gently removed from the mold and stored in a desiccator until further testing or use.

To optimize loading the drug into the MAP tips, five CAB Na hydrogel formulations were developed and used to cast the MAP first layer. The composition of these hydrogel formulations is summarized in Table 2.

### 2.4. MAP Characterization

#### 2.4.1. Determination of CAB Na MAP Drug Content

The patches were first dissolved in 5 mL of deionized water to determine the drug content of the various bilayer CAB Na MAPs. Following complete dissolution, 100 µL of the resulting suspension was transferred to 1.5 mL tubes and mixed vigorously with 0.9 mL of acetonitrile. This process allowed the drug to be completely dissolved and led to the precipitation of water-soluble polymers. The resulting suspension was subsequently centrifuged at 14,800 rpm for 15 min, and 100 µL of the clear supernatant solution was collected and analyzed after making an appropriate dilution with acetonitrile by the validated high-performance liquid chromatography–ultraviolet (HPLC–UV) method [15].

#### 2.4.2. Assessment of CAB Na MAP Mechanical Properties

A TA.XT2 Texture Analyser (Stable Micro Systems, Surrey, UK) in compression mode was used to determine the mechanical properties of the fabricated MAPs, as previously described [24]. Briefly, each MAP was attached to a cylindrical probe (cross-sectional area 1.5 cm^2^), and the texture analyzer arm moved vertically downward at a speed of 0.5 mm/s and the MAP compressed against a flat aluminum block. A force of 32 N was applied for 30 s before the probe moved upward again. The length of the individual needles in each MAP was measured before and after compression using a Leica EZ4 W stereo microscope (Leica Microsystems, Milton Keynes, UK). The percentage reduction of microneedle heights was calculated and reported.

### 2.5. Ex Vivo Drug Deposition Studies

**Figure 3:**
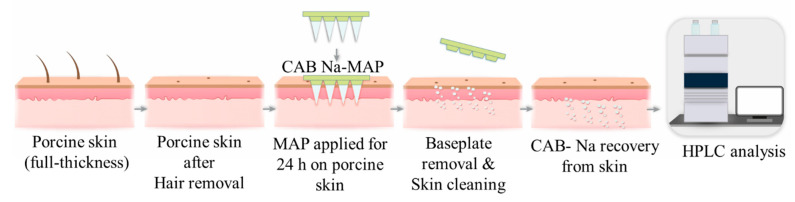
Schematic diagram illustrating the experimental procedures used to evaluate CAB Na deposition ex vivo.

Ex vivo skin deposition studies were conducted to evaluate the efficiency of the MAPs in delivering CAB Na into the skin following administration. These studies were performed using fully excised neonatal pig skin (Figure 3), as described previously [25]. Briefly, the ex vivo skin samples were placed on paper tissue sheets soaked in 10 mM phosphate-buffered saline (PBS) (pH 7.4) solution to maintain skin hydration and placed in a weighing boat. To mimic human skin temperature, the pig skin samples were placed in a preheated incubator for 30 min at 32 °C ± 1 °C. The selected MAPs were subsequently inserted into the skin manually using thumb pressure for 30 s. Afterward, a 13 g cylindrical stainless steel block was placed on top of each MAP to keep it in place, and the skin samples were incubated again at 32 °C ± 1 °C for 24 h. Another weighing boat containing 10 mM PBS (pH 7.4) was placed on top to maintain skin hydration. The inserted MAPs were removed from the skin following 24 h of application, and the skin surface was thoroughly cleaned by applying 3 × 1 mL of PBS (pH 7.4) solution and gently wiping with wet paper tissue. The skin at the MAP application site was then visualized using a Leica EZ4 W stereo microscope and stored in Microcentrifuge tubesat −20 °C until further processing and analysis using the validated HPLC–UV method.

### 2.6. In Vivo Drug Deposition and Pharmacokinetic Studies

In vivo studies were performed using a Sprague Dawley rat model to (i) evaluate MAP delivery efficiency by conducting drug deposition studies in vivo; (ii) evaluate drug pharmacokinetics from the finalized MAPs following single-dose administration; (iii) evaluate the advantage of potentially initiating therapy by administering a loading dose of CAB intramuscularly, followed by MAP application as a maintenance dose; and (iv) assess MAP tolerance and safety.

These in vivo studies were conducted according to the policy of the Federation of European Laboratory Animal Science Associations and the European Convention for the Protection of Vertebrate Animals Used for Experimental and Other Scientific Purposes, following the principles of the 3Rs (replacement, reduction, and refinement). The studies were performed under Institutional Animal Project License number 2903 and Personal License numbers 1747 and 1892 at the Queen’s University Belfast Biological Services Unit after obtaining the required ethical permission from the university’s Animal Welfare and Ethics Review Body.

#### 2.6.1. Rat Dose Calculation

The clinical dose of CAB LA equates to 11.42 mg/kg (800 mg per month administered to a person with an average weight of 70 kg) for the initial dose, or 5.71 mg/kg (400 mg per month administered to a person with an average weight of 70 kg) for the maintenance dose. Here, we used a fixed dose per cohort that best approximated the allometric scaling-based calculated theoretical drug dose (as mg/kg) as required per cohort, rather than using a dose as per the rat’s weight. This was carried out for three reasons: (i) a large number of animals were used in these comprehensive in vivo studies, and numerous blood samples per cohort were collected over prolonged periods, which necessitated conducting the experiments in several consecutive stages, leading to inevitable inter-cohort rat body weight variations; (ii) the difficulty of administering a rat body weight-tailored drug volume from a highly concentrated CAB LA nanosuspension (200 mg/mL), considering the minimum volume that can be accurately administered to a rat using commercially available syringes is 50 µL; and (iii) the difficulty of tailoring MAP drug content per the body weight of each rat, as this could have affected the MAP properties. Therefore, we calculated the IM dose per cohort, assuming that the average weight of a rat aged 12 to 18 weeks is 0.25 kg. For MAP cohorts, four MAPs were applied with the maximum possible dose for the respective MAP design and formulation. 

#### 2.6.2. In Vivo Study Design and Experimental Procedure

Healthy female Sprague Dawley rats (n = 57) aged 12 to 18 weeks were purchased from Envigo Holdings, Inc. (Huntingdon, UK) and allowed to acclimatize to laboratory conditions for at least 7 days before experimental work was commenced. The rats were split randomly into 16 cohorts: 10 cohorts for the in vivo drug deposition study (cohorts 1 through 10; n = 3 rats per cohort) and 6 cohorts for the in vivo pharmacokinetic studies (cohorts 11 through 16; n = 6 rats per cohort). Cohorts 11 through 13 received a single dose of CAB via either MAP or IM injection, and the resulting plasma levels were measured for 28 days. Cohorts 14 through 16 were used in a multidose study, starting with a single loading dose by MAP or IM injection, followed by a maintenance dose either by MAP or IM injection every week for three weeks. A summary of the experimental details for each cohort is reported in Table 3.

For rats that received the drug by MAP application (cohorts 2 through 10, 12, 13, 15, and 16), to minimize interference with the rat’s hair, an electric hair clipper was used to remove the bulk hair, and a depilatory cream was applied to remove any residual hair. To facilitate hair shaving and MAP application, rats were sedated using gas anesthesia (2% to 4% *v*/*v* isoflurane in oxygen). This was performed 24 h before MAP application to allow skin integrity to be restored in the event of being affected by the shaving or hair removal cream. The MAPs were then applied manually to the rat’s back, followed by applying a pressure-sensitive adhesive tape (Microfoam™ Surgical Tape, 3M, Bracknell, UK) and an occlusive dressing film layer (Tegaderm™, 3M, St. Paul, MN, USA). Finally, kinesiology tape (ProWorks Corporation, Corvallis, OR, USA) was used to wrap the backs of the animals to hold the MAPs in place and provide occlusion. The MAPs were applied for 24 h even though the MAP dissolution time was much shorter because anesthesia was needed to remove the MAP, which could not be administered more than once within 24 h, as per the Institutional Animal Project License. Following application for 24 h, the adhesive was removed, and the MAP residuals were gently cleaned by washing the application sites with 1 mL of 10 mM PBS (pH 7.4) solution three times and wiping with prewetted tissue paper.

In the rats used for the in vivo drug deposition studies (cohorts 1 through 10), following 24 h of MAP application, the rats were euthanized, and a blood sample (approximately 200 µL) was collected from each rat in heparin-containing Eppendorf Tubes via cardiac puncture. Additionally, the skin at each MAP application site was harvested individually and stored in Eppendorf Tubes at −80 °C until processing and analysis using the high-performance liquid chromatography-mass spectrometry (HPLC-MS) method [15]. Drug deposition into the in vivo skin was considered cumulatively from the skin-deposited drug and the calculated drug amount from the plasma within 24 h. 

In the rats used for in vivo pharmacokinetic studies, a blood sample (approximately 200 µL) was collected from each rat in Eppendorf Tubes (containing 10 µL of heparin) via tail vein bleeds at predefined time intervals over the study periods as per cohort. Plasma was separated by centrifuging the blood at 5000 rpm for 15 min at 4 °C in a refrigerated centrifuge (Sigma^®^ 2-16 K benchtop refrigerated centrifuge, SciQuip Ltd., Shropshire, UK). Plasma samples were collected into microtubes and stored at −80 °C until processing and analysis using the validated HPLC-MS. The obtained data were used to construct CAB concentration versus time profiles (pharmacokinetic profiles).

### 2.7. Pharmaceutical Analysis

The samples collected from the above-mentioned studies were processed and analyzed as described in the following subsections.

#### 2.7.1. Preparation of Drug-Containing Samples from the In Vitro Studies for Drug Analysis

Samples were processed as previously described [15]. Briefly, samples collected from the in vitro studies were diluted with an appropriate volume of acetonitrile and vortexed (IKA Vortex 2) for 5 min to ensure complete solubilization of the drug, and then centrifuged at 14,800 rpm for 10 min using Eppendorf MiniSpin™ benchtop centrifuges (Fisher Scientific, Leicestershire, UK). The clear supernatant solution was collected and analyzed for drug content using the HPLC–UV method described in Section 2.4.1.

#### 2.7.2. Preparation of Skin Tissue Samples from Ex Vivo Studies for Drug Analysis

To quantify the amount of drug present in biopsied skin samples from the ex vivo drug deposition studies, two stainless steel beads and 500 µL of deionized water were added to each skin sample after being shredded into small pieces, followed by homogenization for 15 min using a TissueLyser LT homogenizer (Qiagen, Hilden, Germany) to solubilize the remaining CAB MAP shafts deposited in the skin. Subsequently, 1 mL of acetonitrile was added to each sample, and the mixture was homogenized for another 15 min to solubilize the drug again. Each sample was then diluted with 3.5 mL of acetonitrile or acetonitrile/water (1:1, *v*/*v*) and vortexed for 5 min. An aliquot of 100 µL of the final skin homogenate was diluted with 900 µL of acetonitrile, vortexed for 30 s, and then centrifuged at 14,800 rpm using an Eppendorf MiniSpin benchtop centrifuge for 10 min. The clear supernatant solution was collected and analyzed for drug content using the validated HPLC–UV method [15].

#### 2.7.3. Preparation of Plasma Samples from In Vivo Studies for Drug Analysis

A total of 300 µL of acetonitrile was added to an aliquot of 100 µL of each plasma sample collected from the in vivo studies (for blood protein precipitation) and vortexed (IKA Vortex 2, at level 6) for 15 min. Samples were then centrifuged at 14,800 rpm for 15 min. Then, 100 µL of supernatant was transferred into an HPLC vial and analyzed for drug content using the previously reported HPLC-MS method [26].

### 2.8. Pharmacokinetic Parameter Calculation

Data collected from analyzing the blood samples from the in vivo studies were used to construct drug concentration versus time profiles for rats in each cohort individually and then as a cohort after calculating the mean ± standard deviation (SD) of drug concentration for each time point. Noncompartmental pharmacokinetic analysis was carried out for each cohort using Microsoft Excel^®^ 2016 (Microsoft Corporation, Washington, DC, USA) with the PKSolver 2 add-in [27], and key pharmacokinetic parameters (as required per cohort) were calculated.

The relative bioavailability (FR) of CAB from MAPs in comparison with IM injection was calculated using the following equation:

(1) $$FR=\frac{{AUC}_{0\text{-}last}(MAP)\times Dose(IM)}{{AUC}_{0\text{-}last}\left(IM\right)\times Dose(MAP)}$$

where AUC_0–last_ (MAP) is the area under the curve (AUC_0–last_) obtained following the finalized bilayer CAB MAP application; AUC_0–last_ (IM) is the area under the curve (AUC_0–last_) obtained following CAB LA administration by IM injection; Dose(IM) is the drug dose administered by IM injection; and Dose(MAP) is the dose administered by MAP.

### 2.9. Statistical Analysis

All results are expressed as the mean ± SD (n ≥ 3), calculated using Microsoft Excel 2013. Statistical analysis was carried out using GraphPad Prism version 6 (GraphPad Software, La Jolla, CA, USA), followed by Tukey’s multiple comparisons post hoc test and *t* test for two-group comparisons. A value of *p* < 0.05 was considered statistically significant.

## 3. Results and Discussion

### 3.1. Characterization of MAP Designs for Improved Insertion and Drug Delivery Performance

The objective of this work was to evaluate various strategies to increase MAP delivery efficiency. These strategies included (i) increasing MAP insertion capability by reducing the MAP needle density from 256 needles per array (D1) to 121 needles per array (D2); (ii) changing the shape of the MAP needles to a full conical shape (D3) or to a full pyramidal shape (D4); (iii) reducing the needle height (D5); and (iv) increasing the length and width of the needles themselves (D6). All the CAB Na-loaded dissolving MAPs displayed sharp microprojections on a clean and smooth baseplate following the outlined fabrication procedure. 

**Figure 4:**
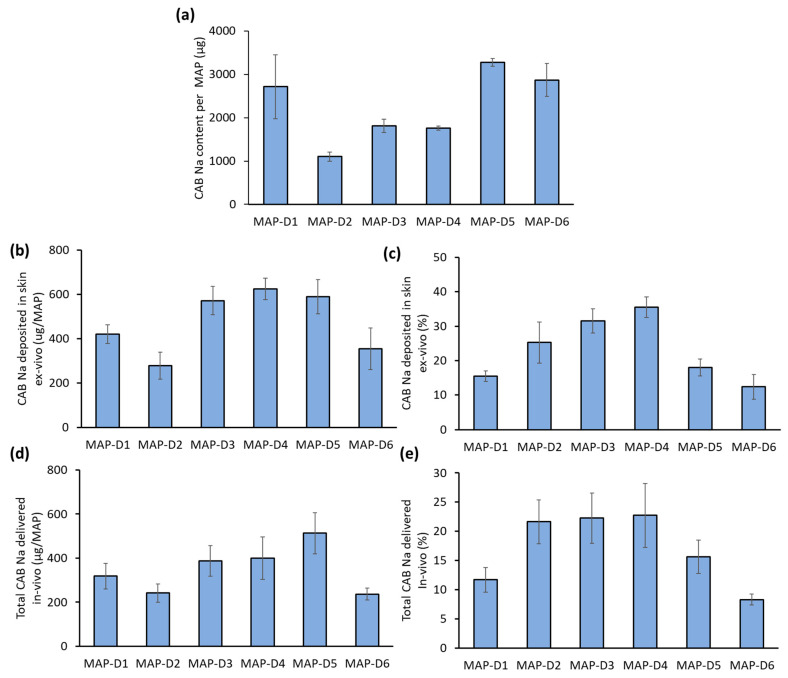
(**a**) Drug loading per patch (µg) for MAPs of different designs and geometries. Data are shown as the mean ± SD (n ≥ 3). (**b**) Amount and (**c**) delivery efficiency of CAB Na into the ex vivo porcine skin following MAP application. Data are shown as the mean ± SD (n ≥ 4). (**d**) Amount and (**e**) delivery efficiency of CAB Na into female Sprague Dawley rats. Data are shown as the mean ± SD (n ≥ 8).

The drug content of each MAP was evaluated following microscopy characterization of the MAPs. Figure 4a shows that the drug content of the patches varied based on the overall design of the patches, as expected. Patch MAP-D2, which had the second lowest number of needles per array (121 per patch), had the lowest drug loading of all the MAP designs: an overall drug loading of 1100 µg per patch. Doubling the number of needles per patch from 121 (MAP-D2) to 256 (MAP-D1) resulted in an increase in the drug loading of the formulation up to approximately 2720 µg per patch. It was observed that the geometry of the needle (full conical MAP-D3 versus full pyramidal MAP-D4) had no significant impact on the overall drug loading of the patch, as the amount of CAB Na loaded into these patches was similar at approximately 1800 µg per patch. It should be noted that increasing the base width of the needles, from 300 µm (MAP-D2) to 500 µm (MAP-D6) also resulted in an increase in MAP drug loading from 1104 µg to 2870 µg per patch, despite having a lower number of needles per patch (81 needles/patch). Next, an ex vivo skin deposition study was conducted to evaluate the ability of these MAPs with different needle geometries and array sizes to deposit CAB Na into the skin. The appearance of the ex vivo neonatal porcine skin following MAP application is shown in Figure S1. Following skin application, the tip of the needle dissolved, resulting in the deposition of the drug into the skin and the formation of white microchannels that were filled with CAB Na drug particles.

Subsequently, the skin samples were harvested, and the amount of drug deposited was quantified. It can be seen in Figure 4b that formulations D3 through D5 had the highest amount of drug deposited into the skin, resulting in an overall deposition of approximately 600 µg of CAB Na into the skin, while formulations D1 and D6 achieved a skin deposition of approximately 400 µg. In contrast, formulation D2 had the lowest skin deposition, amounting to only 280 µg following a single patch application. When this was viewed in terms of delivery efficiency, as shown in Figure 4c, formulations D3 and D4 had the highest percentage delivery efficiencies (approximately 33%) relative to the other formulations. When the amount of drug delivered ex vivo is viewed in terms of amount (µg) and delivery efficiency (%), it can be seen that overall, MAP designs D3 and D4, which consisted of 256 needles per patch and employed either a full conical or a full pyramidal design with a needle length of 850 µm, resulted in the best deposition of CAB Na into the skin. While the amount of drug delivered from D5 was comparable to D3 and D4, the calculated percentage delivery was low due to higher drug loading and the large patch size (0.75 cm^2^) compared to the other designs (0.5 cm^2^).

Nevertheless, considering the physiological and mechanical differences between ex vivo and in vivo skin, it is possible to expect discrepancies in the delivery amounts and efficiencies [28]. Anticipating these discrepancies, we proceeded to evaluate formulations D1 through D6 in vivo using female Sprague Dawley rats. Figure 4d shows that overall, the amount of drug deposited into the skin in vivo followed the same trends but was generally lower for all MAP designs relative to ex vivo skin. Nevertheless, we still observed that MAPs D3 through D5 deposited the highest amount of drug into the rat skin: approximately 400 µg/patch. In contrast, D2 and D6 deposited the lowest amount of drug into the skin, achieving only approximately 240 µg/patch. When the data are viewed in terms of delivery efficiencies (Figure 4e), compared to the other MAP designs, D2, D3, and D4 achieved the highest delivery efficiencies: approximately 22%. The lower amount delivered and delivery efficiencies observed in vivo may be attributed to the reduced insertion depth that was able to be achieved in live animals relative to ex vivo skin samples. In a live animal, the skin is still connected to additional adipose and muscle tissues, which may present some level of resistance (as well as inconsistency in texture/firmness) to the overall insertion of the needle in the array [29]. This reduced insertion depth would result in less drug-containing needle length being inserted into the skin, resulting in a reduction in the amount of drug deposited, which is then reflected in an overall reduced delivery efficiency.

It is worth noting that MAP D6 not only exhibited the lowest amount of CAB Na delivered but also had the lowest delivery efficiency of all six MAP designs. This may be attributed to the large width of the needles, which provided more surface area per needle, resulting in more area of contact with the skin during insertion, which would have caused each needle to encounter more frictional resistance during insertion, resulting in a lower insertion depth. Ultimately, this culminated in lower needle dissolution and drug deposition into the skin. 

MAP D5 exhibited the highest amount of CAB Na delivered into the rat skin in vivo (500 µg/patch) relative to the other patches. This observation was attributed to the fact that there were more needles per patch, enabling more tips to be inserted into the skin, leading to greater deposition of the drug into the skin. Overall, this first part of the study clearly shows that overall MAP geometry as well as the number of needles per array have a profound impact on the delivery of CAB Na into ex vivo and in vivo skin. Moving forward, designs D1, D3, D4, and D5 were subjected to further refinement to enhance delivery efficiency. In contrast, we decided to exclude D2 from further optimization work due to its low needle per array count, which led to the lowest drug loading (Figure 4a). In addition, design D6 was omitted, as this design presented the lowest drug delivery efficiency during the initial screening study. 

### 3.2. Improving the MAP Formulation to Enhance MAP Delivery Efficiency

**Figure 5:**
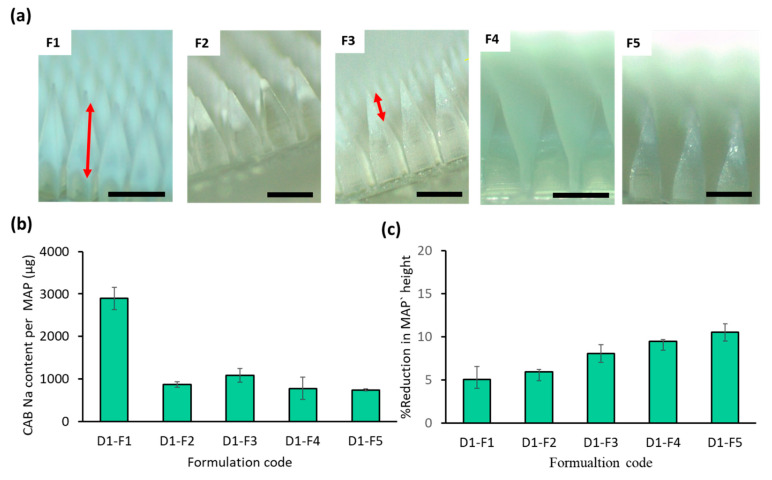
(**a**) Representative light microscope images of MAPs manufactured using diluted CAB Na-hydrogel formulations F1 through F5, with MAP design D1 showing the localization of the drug into the tip (scale bar = 500 µm). Red arrow shows the drug loaded part in the MAP tips (**b**) Drug loading per patch (µg) for MAP-D1 with different hydrogel compositions. Data are shown as the mean ± SD (n ≥ 3). (**c**) Mechanical strength of MAP-D1 prepared by using formulations F1 through F5 (reported as %MAP shaft height reduction after applying a compression force of 32 N against aluminum block following a standardized test using a texture analyzer. Data are reported as mean ± SD (n = 3).

We then evaluated whether we could optimize the drug composition of the needle for each MAP design to localize the drug into the tip of the patch in an attempt to further improve the delivery efficiency of the formulation. One approach was to alter the method by which the drug-gel mixture was cast into the mold (F1 to F2 in Table 2). In F2, the same CAB Na-loaded hydrogel as in F1 was cast into the molds. However, after positive pressure casting the first layer mixture into the molds and removing the excess gel from the molds, the top part of the first layer hydrogel was removed by washing with deionized water. This was undertaken to localize the drug into the tip of the formulation. This would enable only the drug-loaded portion of the tip to be inserted into the skin during application, thereby enhancing the overall delivery efficiency of the formulation [30]. However, it was observed that this method resulted in significant variability in drug loading between patches, as evidenced by microscopic images (Figure 5a) and the large error bar shown in Figure 5b. Given the inconsistency of this approach to localizing the drug into the tip, we explored whether diluting the gel mixture would help to promote drug localization into the tip of the MAP. When the water content of the drug/polymer gel mixture was increased from 1.0 mL to 1.5 mL, the drug content was reduced from 2895 µg to 1085 µg (F1 versus F3) (Figure 5b). However, when the mixture was diluted further (F4 and F5), we observed that the drug content in the patches plateaued at approximately 750 µg/patch. The mechanical characteristics in Figure 5c demonstrate a decrease in the mechanical strength of the MAP when the hydrogel formulation was used with increasing water content. The reduction in strength was directly proportional to the dilution factor. This can be attributed to a lower amount of polymer filling the needle cavity during the first layer casting, caused by the higher water content compared to hydrogel formulation F1. However, it is important to note that all MAP-D1 samples prepared from different formulations still exhibited good mechanical strength, with a height reduction of less than 11%. Among the formulations, F3 displayed an intermediate drug content (37% of F1) and maintained strong mechanical properties (height reduction < 8%). As a result, F3 was chosen as the preferred formulation to proceed with the preparation of other MAP designs. Based on the approach of localizing CAB Na at the tips of the MAPs, formulation F3 yielded the highest drug loading, as depicted in Figure 5b. Conversely, formulations F2, F4, and F5 showed uneven drug distribution in the MAP needle tips, making them unsuitable for reproducible MAP production (Figure 5a). Hence, formulation F3 is considered the most suitable for further MAP preparation and drug deposition studies.

**Figure 6:**
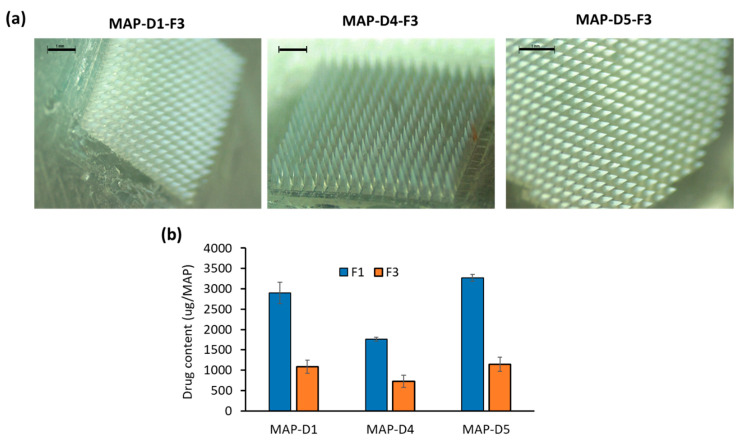
(**a**) Representative light microscope images for the three MAP designs prepared by using formulation F3. (**b**) Comparison of drug content between F1 and F3 for different MAP designs. Data are shown as the mean ± SD (n ≥ 3).

We then proceeded to apply this formulation approach (F3) to the other MAP designs (D4 and D5). The microscopic images in Figure 6a clearly show that selected MAP designs D1, D4, and D5, with hydrogel formulation F1, exhibited complete formation and consistent drug loading across the MAP tips. However, upon changing the formulation from F1 to F3 (Figure 6b), a notable decrease in drug loading within the MAPs was observed for all three selected designs. In general, all MAPs had a drug loading of approximately 0.7 mg to 1.1 mg per patch when F3 was cast into these MAP designs. We did not evaluate MAP-D3 (full conical) any further, as we observed that D3 resulted in poor needle fidelity and integrity post demolding when F3 was cast into this design, as shown in Figure S2.

**Figure 7:**
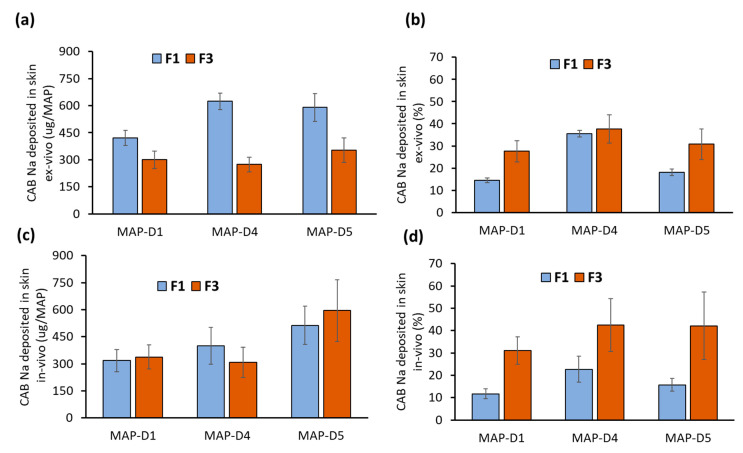
(**a**) Amount and (**b**) delivery efficiency of CAB Na into the ex vivo skin following MAP application. Data are shown as the mean ± SD (n = 5). (**c**) Amount and (**d**) delivery efficiency of CAB Na into female Sprague Dawley rats. Data are shown as the mean ± SD (n = 8).

When the formulation was taken forward into a skin deposition study, it can be seen that, overall, formulation F3 resulted in significantly lower (*p* < 0.05) drug deposition into the skin, as shown in Figure 7a. This was anticipated, as the localization of the drug into the tip resulted in an overall reduction in the drug loading of the patches, which ultimately reduced the amount of drug that could be delivered into the skin. Overall, designs D1, D4, and D5 using formulation F3 resulted in a similar amount of drug deposited into the ex vivo skin: 280 µg to 300 µg. However, when these data were viewed in terms of delivery efficiency, as shown in Figure 7b, we could see that diluting the casted hydrogel via F3 augmented the delivery efficiency of the MAPs. This was most apparent for designs D1 and D5. However, it should be noted that the delivery efficiency of MAP D4 was similar between F1 and F3.

In contrast, when the formulations were taken forward in vivo, we observed that the amount of drug delivered using formulations F3 and F1 was similar (*p* > 0.05), which contradicts the finding that we observed using ex vivo skin. Such discrepancies in results may be attributed to several factors. One is the overhydration of ex vivo skin relative to in vivo skin, which may augment the faster dissolution of MAPs when inserted into ex vivo skin that leads to more expulsion of the drug to the skin surface [31,32].

In this instance, formulation F1, which provided higher drug loading due to the presence of more CAB Na along the needle length, may have undergone partial needle dissolution, mostly in the tip, due to incomplete insertion in vivo. In contrast, as most of the drug was localized in the tips with formulation F3, this portion of the MAP underwent dissolution, which resulted in a similar amount of drug being deposited in vivo. Therefore, when these data are viewed in terms of delivery efficiency, we notice that, overall, F3 had a significantly higher (*p* < 0.05) drug delivery efficiency relative to F1 for designs D1, D4, and D5. Overall, this portion of the work highlights that by diluting the drug/polymer blend that was cast into the molds, we were able to localize the drug into the tip of the MAPs and therefore augment the percentage delivery efficiency of the system, allowing us to reduce the wastage of the undelivered drug.

### 3.3. In Vivo Pharmacokinetic Studies: Combination MAP Designs/Hydrogel Formulation to Enhance MAP Delivery Efficiency

**Figure 8:**
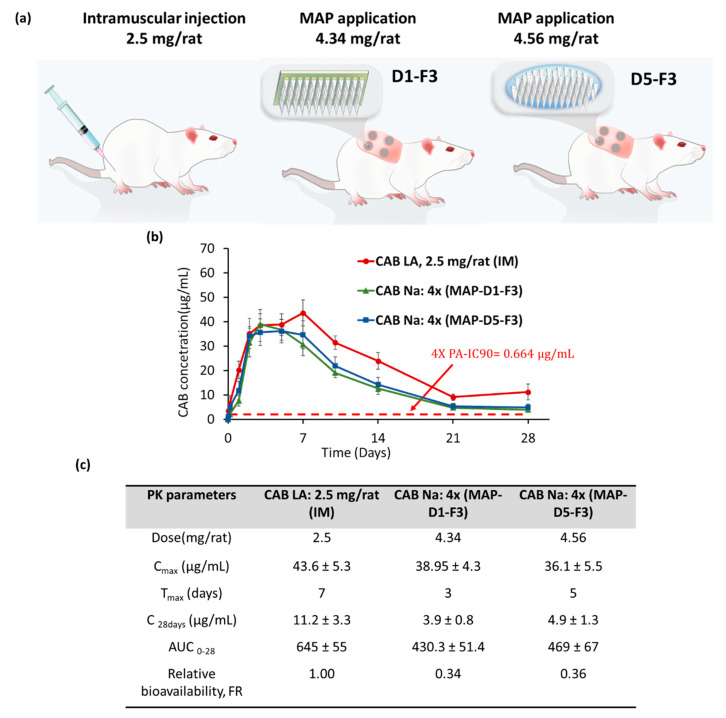
(**a**) Schematic of the pharmacokinetic study design consisting of three different experimental cohorts: (i) rats administered an IM injection at a dose of 2.5 mg/rat; (ii) rats administered MAP-D1-F3 at a dose of 4.34 mg/rat; and (iii) rats administered MAP-D5-F3 at a dose of 4.56 mg/rat. (**b**) Plasma profile of CAB following different experimental strategies. (**c**) Pharmacokinetic parameters for the different experimental groups. Data are shown as the mean ± SD (n ≥ 6).

Guided by this series of work, we proceeded to evaluate the combinatorial effect of formulation F3 with two different MAP designs, D1 and D5, on the pharmacokinetic profile of CAB Na when delivered into Sprague Dawley rats in vivo. In addition, another group of rats, administered an IM injection of CAB Na at a dose of 2.5 mg/animal, was used as a control arm, as illustrated in Figure 8a. It should be noted that although design D4 in combination with formulation F3 exhibited a high delivery efficiency of approximately 40%, this formulation, similar to D3, suffered poor MAP fidelity post demolding. Therefore, sufficient quantities of MAP-D4-F3 could not be manufactured for the pharmacokinetic study; only designs D1 and D5 were taken forward for further formulation characterization in vivo.

When the MAPs were applied as a single dose in vivo, we observed that the plasma level of CAB Na followed a similar trend to that of the IM injection arm. Although the total dose administered to the animal in the MAP experimental groups was much higher (approximately 4 mg/rat) (the delivered dose based on the in vivo skin deposition study was 1.3 mg and 2.4 mg per rat from MAP-D1-F3 and MAP-D5-F3, respectively) than that in the IM injection group (approximately 2.5 mg/rat), the C_max_ values achieved by all the experimental groups were similar (*p* > 0.05) at approximately 40 µg/mL. Nevertheless, we observed that the T_max_ for the MAP experimental group was much earlier relative to the IM injection group, with MAP-D1-F3 displaying a T_max_ of 3 days. This observation may be attributed to the physiochemical properties of the drug injected or applied to the animal. Regarding the IM injection group, CAB was administered as a free acid drug in the form of a nanosuspension. Injection into the highly vascularized muscle tissue allowed the drug to slowly dissolve and diffuse into the rich muscular vasculature, enabling it to reach systemic circulation [33]. However, the drug is a BCS Class II drug, exhibiting good permeability but poor aqueous solubility, and displays a solubility profile that decreases when the surrounding pH is below pH 10 [34]. Given that the pH of normal resting muscle tissue is 5.99 [35], it can be expected that the injected CAB would exhibit very low solubility, causing the drug particle to slowly dissolve from the injection site before reaching systemic circulation. In contrast, when CAB was delivered into the skin via MAPs, the drug was delivered as a sodium salt (CAB Na), which exhibits higher aqueous solubility relative to the free acid, enabling more rapid dissolution from the application site into systemic circulation [26]. Furthermore, this form of administration would generate multiple micron-sized depots from the microneedles inserted. Relative to one large IM depot, the presence of separate and individual micro-depots generated by MAPs would exhibit a larger total surface area, enabling more rapid drug dissolution into systemic circulation [16]. The combination of these effects may serve as a possible explanation for why the MAP group exhibited a more rapid onset of C_max_ relative to the IM injection group.

When examining the in vivo skin deposition, the amount of drug delivered by MAP-D1-F3 was 31% and by MAP-D5-F3 was 42%, which is a percentage of the total dose loaded into the MAP. This pattern was also reflected in the in vivo pharmacokinetic studies. Notably, the area under the curve (AUC) for both MAP groups was significantly lower (*p* < 0.05) when compared to the IM injection group. This reduction in AUC corresponds to the relative bioavailability; upon comparison, it was found that the bioavailability of both MAP groups was 66% less than that of the IM injection group.

This observation was attributed to the incomplete insertion of the MAPs into the skin, as discussed in Section 3.2, which resulted in the incomplete delivery of the dose applied to the skin. This is an occurrence frequently observed when MAPs are applied to the skin in vivo in animals [36,37]. Such an observation is frequently attributed to the elastic nature of the skin, which resists insertion and complete penetration of the MAPs into the skin [38]. Despite this inherent drawback with MAP application, we still observed the capability of MAPs to deliver CAB concentrations to levels exceeding the IC_90_ values. This is of great importance, as therapeutically relevant levels of CAB ought to be greater than IC_90_ to ensure the drug is capable of exerting an efficacious effect in vivo. In addition, when extrapolating these concentrations from a nonhuman model, 4× IC_90_ is usually preferred to account for any physiological differences between species [38]. As shown in Figure 8b, the plasma levels achieved by both MAP groups were well above 4× IC_90_ at 28 days after a single application. 

### 3.4. In Vivo Pharmacokinetic Studies: Repeated Dosing of D1 and D5 Using F3

Finally, we evaluated the effect of a repeated maintenance dose of the final optimized dissolving MAP on CAB plasma levels relative to IM injection. In this part of the study, we evaluated only MAP-D1-F3. Although MAP-D1-F3 and MAP-D5-F3 had similar drug loading and achieved similar pharmacokinetic profiles following a single MAP application, as shown in Figure 6c and Figure 8b, respectively, formulation MAP-D1-F3 displayed more reproducibility in delivery efficiency, as evidenced by the smaller error bar shown in Figure 7d. From a design perspective, MAP-D5-F3 had 600 needles/patch, which is far greater than MAP-D1-F3, which had only 256 needles/patch. Having a greater number of needles per patch may be advantageous in the case of increasing the drug loading of the system. However, in this case, as the drug loading for both systems was similar when casted with F3, having more needles per array would have been disadvantageous. This can be attributed to the system being more difficult to insert into the skin due to the bed of nails effect with more needles per patch [39]. Additionally, from an end-user perspective, the use of a smaller patch design may be advantageous, as this would enable patients to easily apply and insert the patch into the skin with minimal effort and thus provide reassurance that they applied the formulation correctly. Nevertheless, the ease and usability of these different designs from an end-user perspective would warrant a separate human factors evaluation to identify which design would be most preferable for patients.

**Figure 9:**
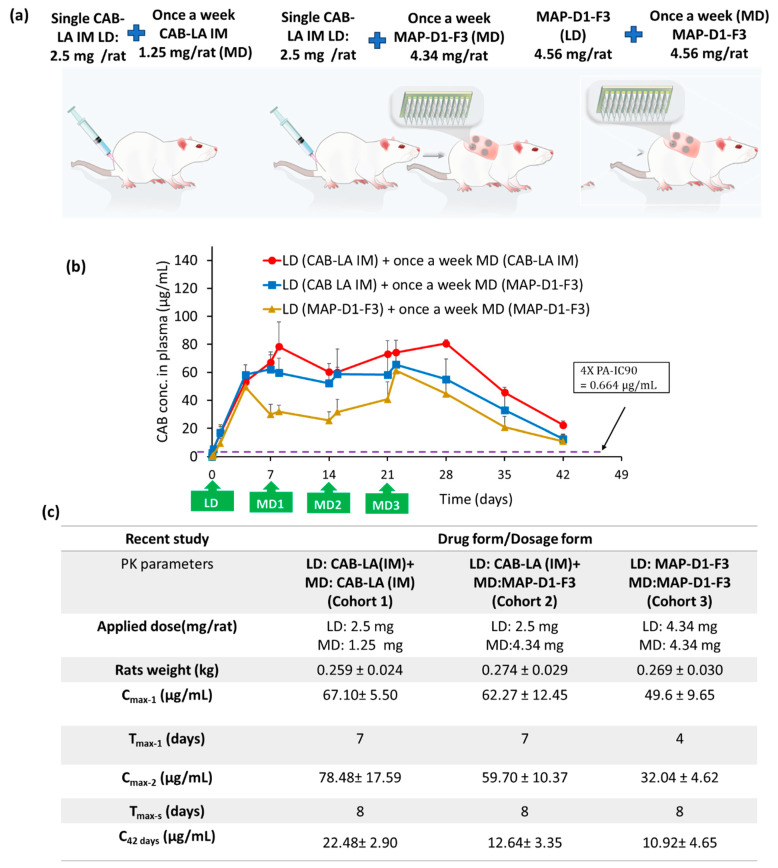
(**a**) Schematic of the pharmacokinetic study design for repeated dosing consisting of three different experimental cohorts: (i) rats administered an IM injection at a loading dose of 2.5 mg/rat, followed by a maintenance dose of 1.25 mg/rat via IM injection once a week; (ii) rats administered a loading dose of 2.5 mg/rat via IM injection, followed by a maintenance dose using a MAP at a dose of 4.34 mg/rat once a week; (iii) rats administered a MAP at a loading dose and a maintenance dose of 4.34 mg/rat once a week. (**b**) Plasma profile of CAB following different administration strategies. (**c**) Pharmacokinetic parameters for the different experimental groups. Data are shown as the mean ± SD (n ≥ 5). C_max-1_ and C_max-2_: first and second maximum plasma concentration, respectively. T_max-1_ and T_max-2_: first and second minimum time for C_max_, respectively.

In the repeated dosing study, MAP-D1-F3 was given once weekly over the course of one month, and the plasma concentration of CAB Na was monitored over the course of 42 days. In this study design, three cohorts were tested: (i) IM injection at a dose of 2.5 mg/rat as both a loading dose and a maintenance dose once weekly; (ii) IM injection at a dose of 2.5 mg/rat as a loading dose, followed by once-weekly MAP application as a maintenance dose; and (iii) MAP application as a loading dose, followed by once-weekly MAP application as a maintenance dose, as shown in Figure 9a. In cohorts 1 and 2, a similar C_max-1_ of approximately 60 µg/mL was achieved on day 7. In contrast, cohort 3 exhibited a more rapid C_max-1_ on day 4 at a lower plasma concentration of approximately 50 µg/mL. When the formulations were administered on a weekly basis, we observed that the plasma concentration of CAB Na managed to achieve pseudosteady-state concentrations after the second dose. Cohorts 1 and 2 achieved pseudosteady-state concentrations of approximately 60 µg/mL, while cohort 3 achieved a much lower steady-state concentration of approximately 30 µg/mL. Although the steady-state concentration achieved by cohort 3 was only half that achieved by cohorts 1 and 2, this concentration was well above 4× IC_90_. Overall, this repeated dosing pharmacokinetic study not only showed that the MAP-only group was able to achieve a more rapid C_max_, but the pseudosteady-state concentrations achieved with repeated applications were well above the required plasma concentration needed to achieve viral suppression. Based on the results shown in Figure 9b, it can be postulated that the patches may need to be administered once a week to achieve physiologically relevant plasma concentrations of CAB. Alternatively, when this result is viewed in tandem with the data shown in Figure 8, it is possible to postulate that the CAB Na-loaded dissolving MAPs could also be administered as a monthly dose. However, the reduction in dosing frequency from once weekly to once monthly would necessitate a larger patch size in humans, which is likely to be larger than would be acceptable to potential users [40,41]. Additionally, the reduction in dosing frequency to once a month may also result in a large dynamic range of CAB plasma levels relative to a weekly patch application.

## 4. Conclusions

Overall, this work highlights the development of a range of dissolving MAPs of different designs and hydrogel compositions loaded with CAB Na. This library was evaluated both ex vivo and in vivo to develop a dissolving MAP that can efficiently deliver CAB Na for the prevention of HIV. This research shows that a combinatorial approach to design and formulation is needed to develop MAPs with tip-localized CAB Na that can aid in augmenting the delivery efficiency of the MAP. A limitation of this work is that when scaled to humans, a very large patch may be required to deliver the dose needed to elicit a protective effect; larger patches would not necessarily be easy to apply and may not be acceptable for end users. Nevertheless, the patches developed in this work could potentially be useful for pediatric patients who require a much lower dose than adults. Alternatively, the study design may be suitable for more potent ARVs that require a lower dose. Further studies are required to identify whether such a formulation strategy is applicable to the novel anti-HIV compounds that are emerging on the market. Based on the results, along with the advancements made within the MAP field, it could be postulated that future MAPs may co-deliver (side by side) several anti-HIV drugs in a single patch that could be used for ARV treatment of HIV-infected patients. Such a composite system would indubitably simplify the complex regimen to which these patients are strictly required to adhere. Nevertheless, before such a system could have a true impact in the clinic, suitable pharmacokinetics and safety must first be demonstrated in humans.

## Figures and Tables

**Table 1:** Different MAP mold designs consisting of different geometries and array sizes.

Code	Design	Length (µm)	Base Width (µm)	Interspacing (µm)	Array Size (cm^2^)
MAP-D1	16 × 16 (256) Cuboidal base/pyramidal tips	900	300	100	0.5
MAP-D2	11 × 11 (121) Cuboidal base/pyramidal tips	900	300	300	0.5
MAP-D3	16 × 16 (256) Full conical	900	300	100	0.5
MAP-D4	16 × 16 (256) Full pyramidal	900	300	100	0.5
MAP-D5	24 × 25 (600) Full pyramidal	700	300	50	0.75
MAP-D6	9 × 9 Cuboidal base/pyramidal tips	1150	500	300	0.5

Abbreviation: MAP, microarray patch.

**Table 2:** Different hydrogel (PVA/PVP/CAB Na/water) compositions cast into the MAP mold.

	Formulation Code				
Composition	F1	F2 *	F3	F4	F5
CAB Na, g	0.84	0.84	0.84	0.84	0.84
PVA (10 kDa) gel (40%*w*/*w*), g	0.6	0.6	0.6	0.6	0.6
PVP K29-32 gel (40%*w*/*w*), g	0.6	0.6	0.6	0.6	0.6
Water, mL	1.0	1.0	1.5	2.0	2.5

Abbreviations: CAB Na, micronized cabotegravir sodium; PVA, poly(vinyl alcohol); PVP, poly(vinyl pyrrolidone). * F2: The same CAB Na-loaded hydrogel formulation as F1; however, following its casting into the MAP mold and removing the excess hydrogel, the top part of the hydrogel was removed by washing the first layer with deionized water.

**Table 3:** Summary of experimental details for each cohort, including rat weight, drug form, administration route, array design, dosing regimen, administered dose per rat, and study period.

Cohort	Administration Route/Array Design/Dosing Regimen	Study Objective	Rat Weight	Number of MAPs or Injection/Rat	Total Dose/Rat (mg)	Study Period (Days)
1	Intradermal/Single dose	Drug deposition	228 ± 8	2 × 50 µL	2.6 mg	1
2	MAP-D1-F1/Single dose		236 ± 4	4 MAP	10.9 mg	1
3	MAP-D2-F1/Single dose		222 ± 4	4 MAP	4.4 mg	1
4	MAP-D3-F1/Single dose		224 ± 9	4 MAP	7.2 mg	1
5	MAP-D4-F1/Single dose		215 ± 3	4 MAP	7.04 mg	1
6	MAP-D5-F1/Single dose		217 ± 7	4 MAP	13.1 mg	1
7	MAP-D6-F1/Single dose		215 ± 6	4 MAP	11.5 mg	1
8	MAP-D1-F3/Single dose		207 ± 5	4 MAP	4.3 mg	1
9	MAP-D4-F3/Single dose		209 ± 21	4 MAP	2.9 mg	1
10	MAP-D5-F3/Single dose		210 ± 12	4 MAP	4.6 mg	1
11	IM/Single dose	Single dose PK study	218 ± 11	50 µL	2.5 mg	28
12	MAP-D1-F3/Single dose		212 ± 8	4 MAP	4.3 mg	28
13	MAP-D5-F3/Single dose		200 ± 6	4 MAP	4.6 mg	28
14	LD: IM MD: IM	Repeated dose PK study	259 ± 24	LD: 50 µL MD: 50 µL	LD: 2.5 mg MD: weekly 1.25 mg	42
15	LD: IM MD: MAP		274 ± 29	LD: 50 µL MD: 4 MAP	LD: 2.5 mg MD: weekly 4.3 mg	42
16	LD: MAP MD: MAP		269 ±30	LD: 4 MAP MD: 4 MAP	LD: 4.3 mg MD: weekly 4.3 mg	42

Abbreviations: IM, intramuscular; LD, loading dose; MAP, microarray patch; MD, maintenance dose; PK, pharmacokinetic.

## Data Availability

All data are included in this manuscript and its Supplementary Files.

## Supplementary Materials

The following supporting information can be downloaded at: https://www.mdpi.com/article/10.3390/pharmaceutics16010142/s1, Figure S1: Appearance of the skin following the application of the MAP on ex vivo neonatal porcine skin. Figure S2: Representative light microscope images for MAP design D4 manufactured using diluted CAB Na-hydrogel formulation F3. The images show the formation of MAPs with poor needle fidelity post demolding, highlighting the incompatibility of formulation F3 with this MAP design. Insufficient quantities of this MAP design with formulation F3 need to be taken forward into an in vivo pharmacokinetic study.
