# Supplementary material for: A Bilayer Microarray Patch (MAP) for HIV Pre-Exposure Prophylaxis: The Role of MAP Designs and Formulation Composition in Enhancing Long-Acting Drug Delivery
Source: Pharmaceutics. 2024 Jan 20;16(1):142. doi: 10.3390/pharmaceutics16010142 (PMC10819247; doi:10.3390/pharmaceutics16010142)
Supplement: Supplementary file 1 [file pharmaceutics-16-00142-s001.zip › supplementary figures.pdf]

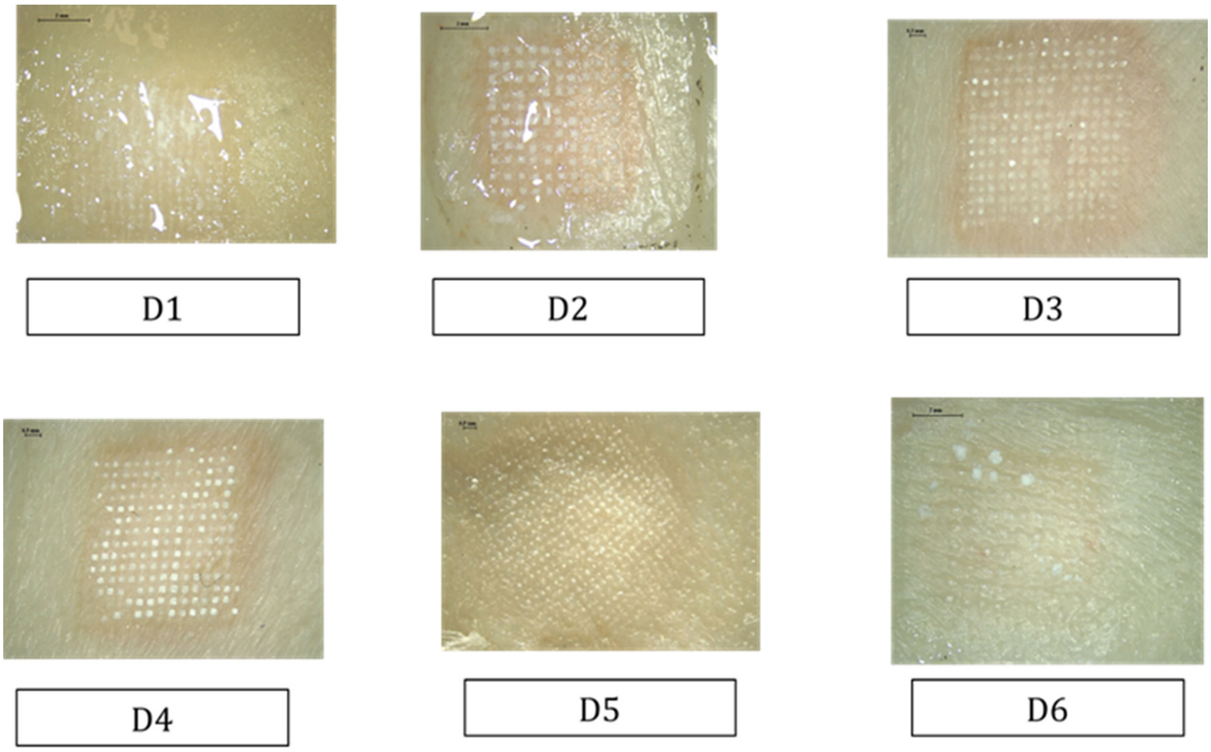

Figure S1. Appearance of the skin following the application of the MAP on *ex vivo* neonatal porcine skin.

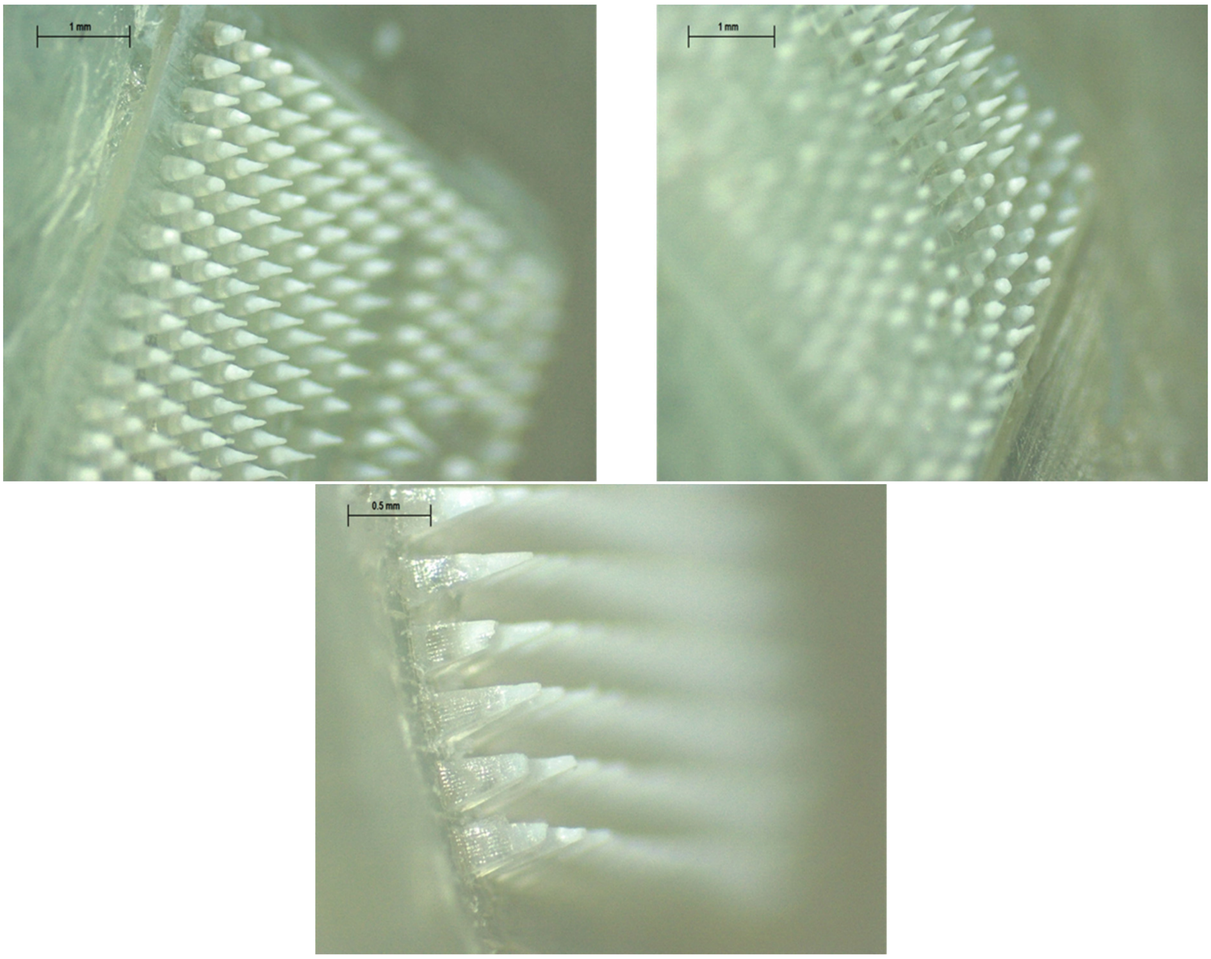

Figure S2. Representative light microscope images for MAP design D4 manufactured using diluted CAB Na-hydrogel formulation F3. The images show the formation of MAPs with poor needle fidelity post demolding, highlighting the incompatibility of formulation F3 with this MAP design. Insufficient quantities of this MAP design with formulation F3 need to be taken forward into an *in vivo* pharmacokinetic study.
